# Commentary: Use of BACTRAC Proteomic Database-Uromodulin Protein Expression During Ischemic Stroke

**Published:** 2021-03

**Authors:** Gabriella-Salome K. Armstrong, Jacqueline A. Frank, Christopher J. McLouth, Ann Stowe, Jill M. Roberts, Amanda L. Trout, Justin F. Fraser, Keith Pennypacker

**Affiliations:** 1 Department of Neurology, University of Kentucky, Lexington, Kentucky, USA; 2 Department of Neurosurgery, University of Kentucky, Lexington, Kentucky, USA; 3 Department of Radiology, University of Kentucky, Lexington, Kentucky, USA; 4 Department of Neuroscience, University of Kentucky, Lexington, Kentucky, USA; 5 Department of Behavioral Science, University of Kentucky, Lexington, Kentucky, USA; 6 Center for Advanced Translational Stroke Science, University of Kentucky, Lexington, Kentucky, USA

**Keywords:** Ischemic Stroke, Uromodulin protein, High Body Mass Index (BMI), Age, Blood and Clot Thrombectomy Registry and Collaboration (BACTRAC)

## Abstract

**Introduction:**

Uromodulin (UMOD) is a glycoprotein expressed by the epithelial cells of the thick ascending limb of Henle’s loop in the kidney. Research has shown that increased uromodulin expression may be associated with lower risk of cardiovascular disease in adults. Utilizing the Blood and Clot Thrombectomy Registry and Collaboration (BACTRAC) (clinicaltrials.gov NCT03153683), a continuously enrolling tissue bank, we aimed to examine the associations between serum uromodulin, age, and high BMI (BMI>25) and its relationship to stroke in patients.

**Methods:**

Arterial blood distal and proximal to the thrombus was collected during a thrombectomy procedure using the BACTRAC protocol and sent to Olink (Boston, MA) to determine proteomic expression via proximity extension assay. Uromodulin expression was recorded and analyzed using two tailed T-tests and linear regressions.

**Results:**

The relationship between systemic and intracranial uromodulin, age, high BMI and hypertension were assessed. Systemic and intracranial uromodulin decreased with age (p<0.0001 and r^2^=0.343, p=0.0416 and r^2^=0.102) respectively. Systemic uromodulin expression increased with BMI>25 (p=0.014). Presence of hypertension decreased uromodulin’s expression systemically (p=0.018) and intracranially (p=0.007).

**Conclusions:**

Uromodulin was increased significantly in overweight patients, decreased significantly in older patients, and decreased in patients with hypertension. The increase in uromodulin in people with high BMI could be a protective reaction of the kidney to worsening conditions that make ischemic stroke more likely, with a goal of delaying dangerous outcomes. The decreased expression of uromodulin in older adults could be associated with the decline of general kidney function that accompanies aging. Hypertension can contribute to an AKI by decreasing perfusion to the kidney, therefore decreasing kidney function and uromodulin production. Further analyses are needed to understand the role of uromodulin following ischemic stroke.

## Introduction

The Blood and Clot Thrombectomy Registry and Collaboration (BACTRAC) (clinicaltrials.gov NCT03153683), a continuously enrolling tissue bank at the University of Kentucky, collects arterial blood distal and proximal to the thrombus from stroke patients undergoing thrombectomy [1]. Plasma proteins from these samples have been isolated and analyzed to create a proteomic database [2]. This database can be utilized to determine proteomic associations in stroke by linking the protein’s expression to patient’s demographics and clinical outcomes. This biobank has an enormous amount of information and numerous uses that are not limited to stroke pathology. This biobank can be used for broad purposes such as protein expression and its use as clinical biomarkers. For this study, uromodulin (UMOD), was chosen to determine its association with patient demographics and clinical outcomes to highlight the multi-faceted uses of the biobank. Uromodulin, also known as Tamm-Horsfall protein, is a glycoprotein that is expressed by the epithelial cells of the thick ascending limb of Henle’s loop in the kidney. It is excreted in the urine and is the main component of hyaline urinary casts. Uromodulin is the most abundant urinary protein in healthy individuals, and has important roles in ion transport, water and electrolyte balance, and prevention of urinary tract infections [3]. Reduced levels of urinary uromodulin have been associated with acute tubular necrosis, diabetic nephropathy, active lupus nephritis, mortality and cardiovascular disease [4,5]. Most studies of uromodulin have focused on utilizing urinary uromodulin and less is known about serum uromodulin. Recently, there has been a revival of interest in studies with serum uromodulin as more assays have become available. Research has shown that increased uromodulin expression may be associated with lower risk of cardiovascular disease in adults [5]. One study showed that higher serum uromodulin concentration was associated with a beneficial metabolic profile, lower prevalence rates of arterial hypertension, diabetes mellitus, heart failure and a lower risk for 10-year mortality [3]. Another study demonstrated an inverse association of serum uromodulin with arterial hypertension in a large population-based study [6]. Utilizing the proteomic data base from BACTRAC, we aimed to analyze uromodulin in the setting of large vessel occlusion stroke to understand the relationship between its intravascular levels and specific elements of stroke such as age, increased BMI (BMI>25), and other common comorbidities tied to increased stroke.

## Materials & Methods

This study evaluated 42 adult subjects (>18yrs), of which 31 were female (57%), average age of 66.8 ± 16.7, average BMI of 25.7 ± 6.7, and 31 (74%) of them had self-reported documented hypertension. During a mechanical thrombectomy, arterial blood distal and proximal to the thrombus was collected using the previously described BACTRAC protocol [1] and sent to Olink (Boston, MA) to determine proteomic expression via proximity extension assay. Uromodulin expression was recorded and analyzed using Welch’s two tailed T-tests and linear regressions. P-value of 0.05 was considered significant. Systemic uromodulin was the uromodulin expression found in arterial blood proximal to the thrombus. Intracranial uromodulin was the uromodulin expression found in arterial blood distal to the thrombus.

## Results

The relationship between systemic and intracranial uromodulin, high BMI, age and hypertension were found significant. Figures 1a and 1b show that systemic and intracranial uromodulin decreased with age (p<0.0001 and r^2^=0.343, p=0.0416 and r^2^=0.102). Figure 2a shows systemic uromodulin expression increased with BMI>25 (p=0.0139) while figure 2b shows no significant change in intracranial expression. The relationship between systemic and intracranial uromodulin expression and the presence of acute ischemic stroke were assessed. Figures 3a and 3b show that systemic and intracranial uromodulin expression decreased with hypertension, p=0.0177 and p=0.0066 respectively.

## Discussion

In our study, uromodulin was found to be increased significantly in overweight patients (BMI>25). In 2017-2018, the prevalence of obesity in adults was 42.4% [7], highlighting the prevalence of a major risk factor for stroke. Research has shown that increased uromodulin expression levels has been associated with lower cardiovascular disease in adults [5] and we suggest that the increase in uromodulin in people with high BMI could be a protective reaction of the kidney to worsening conditions (i.e. overweight and obesity) that make ischemic stroke more likely. Studies have indicated that the prevalence of hyperfiltration, a precursor to chronic kidney disease, is higher among individuals with obesity and this could serve as an alternative explanation to our findings of increased UMOD expression in those that have a high BMI [8].

Uromodulin was found to be decreased significantly in older patients. Aging is one of the most powerful non-modifiable risk factors for stroke and the incident for stroke doubles every 10 years after age 55 [9]. The decreased expression of uromodulin in older adults could be associated with the decline of general kidney function that accompanies aging. Uromodulin is produced exclusively in the kidney and as kidneys age, production of uromodulin can decrease as well. Essentially as individuals’ age, so do their kidneys. Following a stroke, variations in blood pressure and salt wasting, as well as treatments and explorations, can all have the possibility to contribute to the development of an acute kidney injury (AKI) [10]. An AKI can decrease kidney function which can include a decrease in uromodulin production, explaining the decreased levels of uromodulin expression in those that experienced a stroke.

Uromodulin was found to be decreased in patients with hypertension compared to patients without hypertension which is a similar finding to a study done in 2020 demonstrating an inverse relationship with serum uromodulin and arterial hypertension [6]. Hypertension is prevalent in acute kidney injuries and maintaining an ideal blood pressure is important in order to prevent an AKI [11]. Hypertension can contribute to an AKI by decreasing perfusion to the kidney, therefore decreasing kidney function and uromodulin production. Further analyses of clinical data are needed to understand the role of uromodulin after ischemic stroke.

## Conclusion

Utilizing the proteomic data base from BACTRAC, we aimed to analyze uromodulin in the setting of large vessel occlusion stroke to understand the relationship between its intravascular levels and specific elements of stroke such as age, high BMI (BMI>25), and other comorbidities. In our study, uromodulin was found to be increased significantly in overweight patients, decreased significantly in older patients and decreased in those patients with hypertension. This exercise depicts the utility of the BACTRAC proteomic database in which 184 proteins have been analyzed in intracranial and systemic blood from patients undergoing thrombectomy. Any one of these 184 proteins can be selected, and its expression can be associated with clinical outcomes and patient demographics. These results not only elucidate a protein’s role in stroke but also its demographic expression. The limitations to this study include the relatively small sample size of the population and restricted diversity of the patient population. In the future, other institutions will be collecting and analyzing these blood samples to provide greater diversity and sample size which will give insight into plasma protein function during stroke and their relationship to patient demographics.

## Figures and Tables

**Figure 1a:**
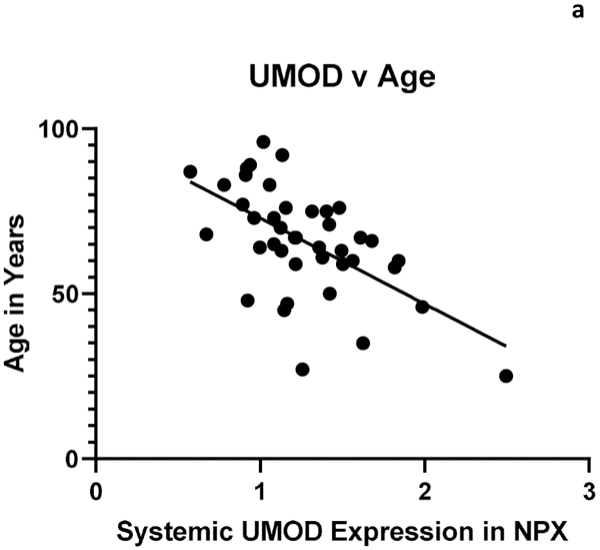
Uromodulin protein plasma expression is decreased in systemic arterial blood with age (p<0.0001 and r^2^=0.343).

**Figure 1b:**
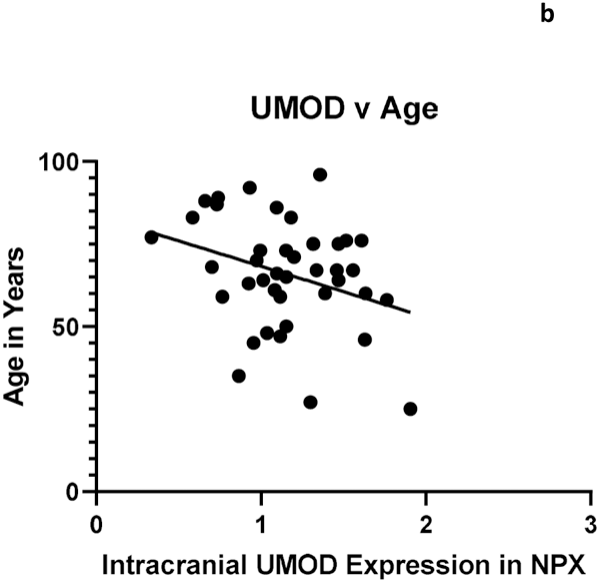
Uromodulin protein plasma expression is decreased in intracranial arterial blood with age (p=0.0416 and r^2^=0.102).

**Figure 2a:**
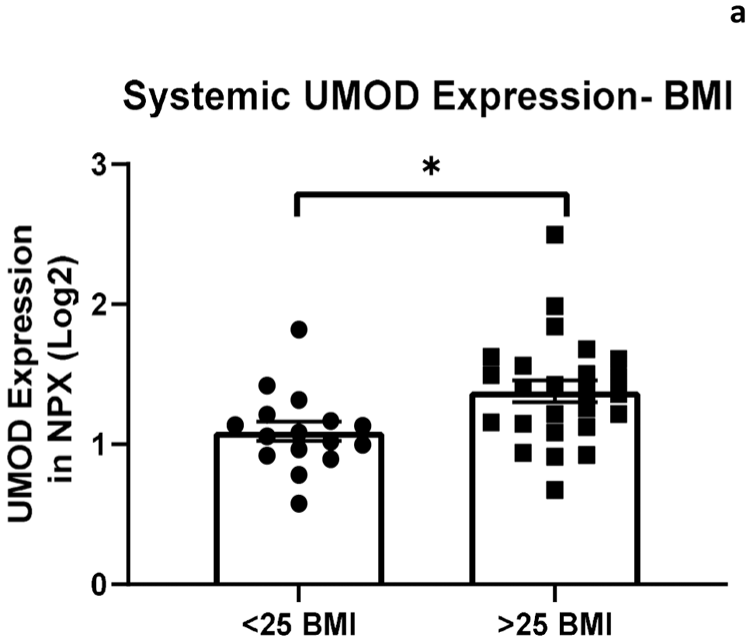
Uromodulin protein plasma expression is increased in systemic arterial blood with BMI greater than 25 (p=0.0139).

**Figure 2b:**
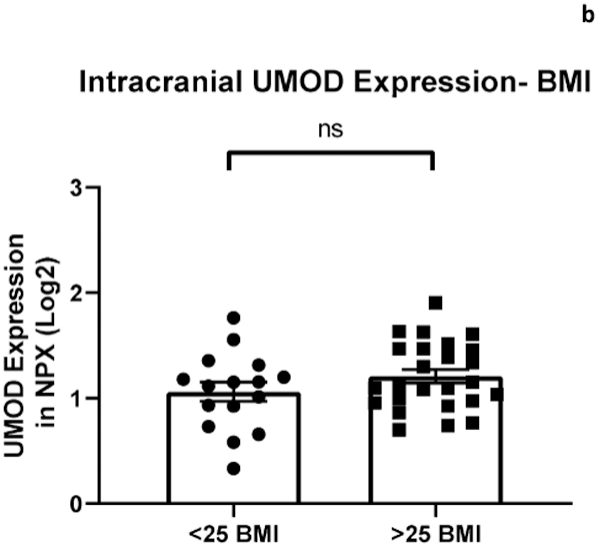
Uromodulin protein plasma expression showed no significant change in intracranial arterial blood based on BMI separated as less than 25 and greater than 25.

**Figure 3a:**
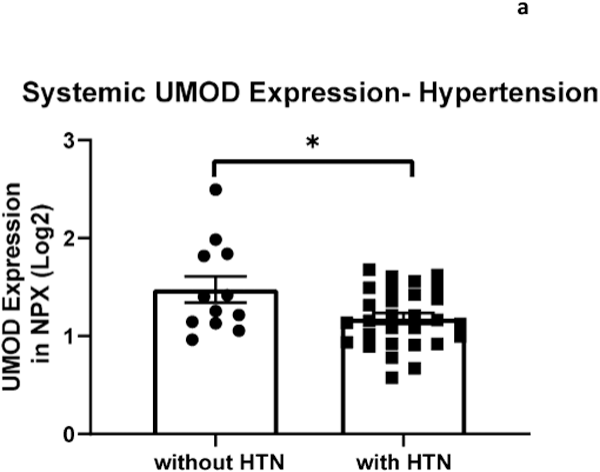
Uromodulin protein plasma expression is decreased in systemic arterial blood in patients with hypertension (p=0.018).

**Figure 3b:**
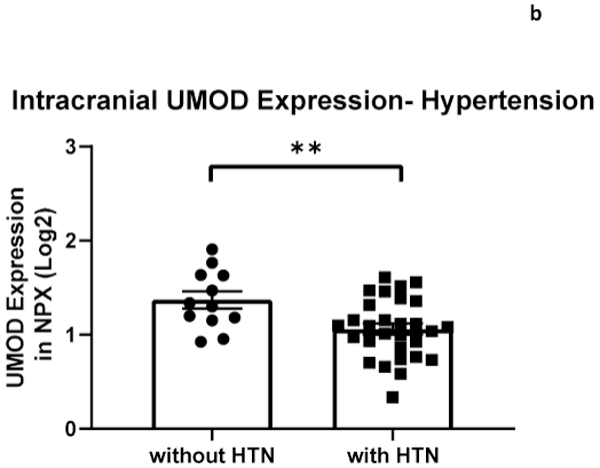
Uromodulin protein plasma expression is decreased in intracranial arterial blood in patients with hypertension (p=0.0066).
